# A Serotonin Transporter Gene (SLC6A4) Polymorphism Is Associated with Reduced Risk of Irritable Bowel Syndrome in American and Asian Population: A Meta-Analysis

**DOI:** 10.1371/journal.pone.0075567

**Published:** 2013-09-19

**Authors:** Mohammed Y. Areeshi, Shafiul Haque, Aditya K. Panda, Raju K. Mandal

**Affiliations:** 1 Department of Medical Microbiology, College of Nursing and Allied Health Sciences, Jazan University, Jazan, Saudi Arabia; 2 Department of Biosciences, Jamia Millia Islamia (A Central University), New Delhi, India; 3 Department of Infectious Disease Biology, Institute of Life Sciences, Bhubaneswar, Odisha, India; 4 Department of Urology, Sanjay Gandhi Post Graduate Institute of Medical Sciences, Lucknow, Uttar Pradesh, India; National Institutes of Health, United States of America

## Abstract

**Aim:**

Association studies of serotonin transporter gene SLC6A4 I/S polymorphism and irritable bowel syndrome (IBS) have shown inconsistent and contradictory results among different populations. In the present study, meta-analysis was performed to evaluate the association between SLC6A4 I/S polymorphism and IBS susceptibility.

**Methodology:**

Systemic assessment was performed for the published studies based on the association of SLC6A4 I/S polymorphism and IBS risk from PubMed (Medline), EMBASE search. A meta-analysis was done to appraise the said association. Pooled odds ratios (ORs) and 95% confidence intervals (CIs) were calculated for allele contrast, homozygous, heterozygous, dominant and recessive genetic model.

**Results:**

A total of twelve studies comprising 2068 IBS cases and 2076 controls were included in this meta-analysis. Overall, no significant results were obtained for S allele carrier (S vs. I: p=0.488; OR=1.073, 95% CI=0.879 to 1.311) Co-dominant (SS vs. II; p=0.587; OR=1.112, 95% CI=0.758 to 1.631), (IS vs. II; p=0.361; OR=0.878, 95% CI=0.665 to 1.160). Similarly, dominant (SS+IS vs. II: p=0.853; OR=0.974, 95% CI=0.736 to 1.288) and recessive (SS vs. II+IS: p=0.267; OR=1.172, 95% CI=0.886 to 1.522) genetic models did not demonstrate risk. In the subgroup population based analysis, reduced risks were found in American (IS vs. II: p=0.009; OR=0.685, 95% CI=0.516 to 0.908) and Asian (SS+IS vs. II; p=0.001; OR=0.116, 95% CI=0.068 to 0.197) population. However, no risk was observed in European population.

**Conclusions:**

This investigation clearly demonstrates that SLC6A4 (Ins/Del) polymorphism is associated with reduced risk of IBS in American and Asian population. However, future well-designed studies with stratified case control and biological characterization will be needed to validate this finding.

## Introduction

Irritable bowel syndrome (IBS) is a predominant and common chronic gastrointestinal (GI) disorder, associated clinical manifestations are recurrent abdominal pain or discomfort, swelling, and altered bowel function, such as constipation, irregular stool frequency and alternation between these two symptoms [1,2]. The pathophysiology of IBS is still unclear, due to recent advancements in biomedical research, now it has been acknowledged that the gene discovery of IBS would greatly aid the global control of this chronic disease as the diagnostic tests are lacking. Earlier reports have already suggested the involvement of serotonin [5-hydroxytryptamine (5-HT)] and reduced level of mucosal serotonin in IBS patients [3,4,5]. The serotonin transporter protein (SERT) or 5-HT transporter (5-HTT) encoded by SLC6A4 gene (GenBank NM_001045) maps on chromosome 17q11, 1q12, mainly responsible for the reuptake of serotonin into mucosal epithelial cells and enteric neurons [6]. SERT is a significant neurotransmitter and paracrine signaling molecule in the gut [7]. SERT signaling plays an important role in the modulation of brain-gut communication and functional gastrointestinal disorders [7]. The expression of SERT gene is regulated by a complex transcriptional activity and can be inﬂuenced by genetic polymorphism [8]. It could be possible that individuals with IBS have a genetic predisposition for decreased or increased SERT expression. Abnormalities in serotonin reuptake can alter enteric serotonergic signaling, leading to gut dysfunctions that are involved in the pathophysiology of IBS [9]. These studies highlighted the significance of serotonin in IBS disease.

Several polymorphisms have been discovered in this gene, among them polymorphism of a 44-base pair insertion (Ins)/deletion (Del) in the 5’-ﬂanking promoter region (5-HTTLPR), which results in either a long (l) or short (s) transcript has been identified as important one. Homozygous (S/S) and heterozygous (I/S) genotypes have been shown to be associated with lower transcriptional activity compare with of the homozygous long (I/I) genotyping in transfected cell line, and thus lower levels of SERT mRNA and consequently 5-HT reuptake [10,11]. The long variant is associated with high rate of 5-HT uptake, and high level of 5-HT binding in platelets [12].

Having known the functional significance of the serotonin signaling in the gastrointestinal tract, it has been considered that SLC6A4 polymorphism may be a potential susceptibility factor for IBS. Till now, a number of studies have been conducted in different populations but the results are conflicting rather than conclusive, because some studies report association between IBS and SLC6A4 (Ins/Del) polymorphism, whereas others did not show statistical relationship between them [13-24]. Inconsistency in results can be attributed to sample size and ethnic diversity, and individual studies may have low power to detect overall effects. Meta-analysis is a useful tool for investigating the risk factors of any disease by employing quantitative approach, where individual sample sizes are small and low statistical power [25]. Through pooling studies together according to their characteristics, we conducted this meta-analysis based on published literature in peer reviewed journals to make a more comprehensive and compelling evaluation of the connection between SLC6A4 polymorphism (Ins/Del) and IBS risk.

## Materials and Methods

This meta-analysis was conducted according to the standard guidelines of observational studies in epidemiology [26].

### Identification and eligibility of relevant studies

We performed a PubMed (Medline), EMBASE search covering all research papers published with a combination of the following key words: “SERT, SLC6A4, 5-HTT gene, Serotonin transporter gene (polymorphism OR mutation OR variant) AND irritable bowel syndrome or IBS (last updated on March 2013). The references provided in the retrieved publications were also reviewed in case any relevant study was missed. Potentially relevant genetic association studies were evaluated by examining their titles and abstracts, and all published studies matching with the above said key words were retrieved.

### Inclusion and exclusion criteria

In order to minimize heterogeneity and facilitate the proper interpretation of our results, studies included in the current meta-analysis had to meet the following criteria: a) evaluated the association between Ins/Del I>S and IBS risk, b) used a case-control design, c) recruited pathologically confirmed IBS patients and healthy controls, d) have available all three genotype frequencies in cases and controls, e) published in English language. The major reasons for exclusion of studies were, a) overlapping data, b) case-only studies and review articles, and c) genotype frequencies or number not reported. The supporting PRISMA 2009 Flow Diagram (showing identification and selection of studies) is available as supporting information; see Figure S1.

### Data extraction and quality assessment

For each publication, the methodological quality assessment and data extraction were independently abstracted in duplicate by two independent investigators using a standard protocol and data-collection form according to the inclusion criteria listed above to ensure the accuracy of the collected data. In case of disagreement on any item of the data, the problem was fully discussed to reach a consensus. Characteristics abstracted from the studies included the name of the first author, year of publication, the country of origin, the number of cases and controls, types of study, genotype frequencies. These characteristics were important for representing the relevant published studies on the said topic. Also, these characteristics reflect the important features of the included published reports and help in retrieval and utilization of these reports for relevant or similar studies.

### Statistical analysis

Pooled crude ORs and their corresponding 95% CIs were calculated to evaluate the association between SLC6A4 Ins/Del polymorphism and IBS risk. Heterogeneity assumption was appraised by the chi-square based Q-test [27]. Significance level (p-value) > 0.05 for the Q-test indicated a lack of heterogeneity among the studies. Pooled ORs were calculated either by the fixed effects model [28], or by the random effects model [29]. In addition I^2^ statistics was also used to quantify inter-study variability and larger values indicated an increasing degree of heterogeneity [30]. Hardy-Weinberg equilibrium (HWE) in the control group was measured via chi-square test. Funnel plot asymmetry was evaluated by Egger’s linear regression test which a linear regression methodology to measure the funnel plot asymmetry on the natural logarithm scale of the OR. The significance of the intercept was determined by the t-test (p-value < 0.05 was considered as a representation of statistically significant publication bias) [31]. Overall, as well as subgroup analysis stratified by the study type or participants’ region or race were performed to evaluate the association in different aspects. The statistical analysis for this meta-analysis was performed with the help of comprehensive meta-analysis (CMA) V2 software (Biostat, USA).

## Results

### Characteristics of the published studies

A total of thirty six articles were achieved by literature search from the PubMed (Medline) and EMBASE web portal databases (Figure S1). All retrieved articles were examined by reading the titles and abstracts, and the full texts for the potentially relevant publications were further checked for their suitability for this meta-analysis. In addition to the database search, the reference lists of the retrieved articles were also screened for other potential articles. Studies either using SLC6A4 polymorphism to predict survival in IBS patients or considering SLC6A4 variants as an indicator for response to therapy were excluded. Studies investigating the levels of SLC6A4 mRNA or protein expression or review article were also excluded. Only case-control or cohort design studies having frequency of all three genotype were included. After careful screening and following the inclusion and exclusion criteria, twelve eligible original published studies were found and included in this meta-analysis (Supporting information; see Figure S1). Distribution of genotypes, HWE p-value in the controls and susceptibility to IBS are tabulated in Table 1.

**Table 1:** Main characteristics of all studies included in the meta-analysis.

**First Authors**	**Year**	**Country of origin**	**Population type**	**Study design**	**Genotyping method**	**Controls**	**Cases**	**Genotype (Controls**)				**Genotype (Cases**)				**HWE**
								**I/I**	**I/S**	**S/S**	**MAF**	**I/I**	**I/S**	**S/S**	**MAF**	**p-value**
Pata et al. [13]	2002	Turkey	Europe	HB	PCR	91	54	22	34	36	0.57	8	28	18	0.59	0.016
Yeo et al. [14]	2004	USA	America	CT	PCR, Sequencing	429	186	138	214	77	0.42	63	62	61	0.49	0.70
Kim et al. [15]	2004	USA	America	PB	PCR, Sequencing	120	256	31	49	20	0.44	114	136	50	0.39	0.93
Park et al. [16]	2006	Korea	Asia	HB	PCR	437	190	25	139	273	0.78	2	54	134	0.84	0.19
Li et al. [17]	2007	China	Asia	HB	PCR	96	87	7	34	55	0.75	14	27	46	0.68	0.58
Saito et al. [18]	2007	USA	America	HB	PCR	53	41	18	22	13	0.45	14	25	11	0.47	0.23
Kohen et al. [19]	2009	USA	America	PB	PCR	48	185	10	29	9	0.48	69	81	35	0.40	0.14
Sikander et al. [20]	2009	India	Asia	HB	PCR	100	151	17	53	30	0.56	28	71	52	0.57	0.43
Niesler et al. [21]	2010	UK	Europe	HB	PCR	92	390	27	43	22	0.47	136	194	60	0.40	0.54
Markoutsaki et al. [22]	2011	Greece	Europe	HB	PCR	238	124	16	87	135	0.75	4	35	85	0.82	0.69
Wang et al. [23]	2012	China	Asia	HB	PCR, Sequencing	120	254	36	35	49	0.55	90	67	97	0.51	0.001
Kumar et al. [24]	2012	India	Asia	HB	PCR, Sequencing	252	150	46	114	92	0.59	17	44	89	0.74	0.30

Note: CT- Clinical trial; HB- Hospital based; PB- Population based, MAF- Minor allele frequency; HWE- Hardy Weinberg equilibrium

### Publication bias

Begg’s funnel plot and Egger’s test were performed to assess the publication bias among the included studies for the meta-analysis. The appearance of the shape of funnel plots was seemed symmetrical and the Egger’s test was performed to provide statistical evidence of funnel plot. The results showed lack of publication bias (Table 2).

**Table 2:** Overall statistics to test publication bias and heterogeneity in the meta-analysis.

**Comparisons**	**Intercept**	**Egger’s regression analysis**		**Heterogeneity analysis**			**Model used for the meta-analysis**
		**95% CI**	**p-value**	**Q-value**	**P_heterogeneity_**	**I^2^ (%**)	
S vs. I	-2.39	-8.19 to 3.19	0.39	40.85	<0.0001	73.07	Random
SS vs. II	0.20	-3.40 to 3.80	0.90	32.69	0.001	66.35	Random
IS vs. II	2.05	-0.21 to 4.38	0.07	20.07	0.044	45.20	Random
SS+IS vs. II	1.58	-0.98 to 4.14	0.19	23.76	0.014	53.71	Random
SS vs. II+IS	-4.25	-8.21 to -0.28	0.03	37.74	<0.0001	70.85	Random

### Test of heterogeneity

Q-test and I^2^ statistics were used to test for heterogeneity among the included studies. For overall analysis, heterogeneity was observed in all the models, such as allele (S vs. I), homozygous (SS vs. II), heterozygous (IS vs. II), dominant (SS+IS vs. II) and recessive (SS vs. II+IS) genotype model, which were included for the analysis. Thus, random effect model was applied to calculate the pooled ORs and 95% CI (Table 2).

### Overall analysis of SLC6A4 Ins/Del polymorphism and IBS susceptibility

**Figure 1:**
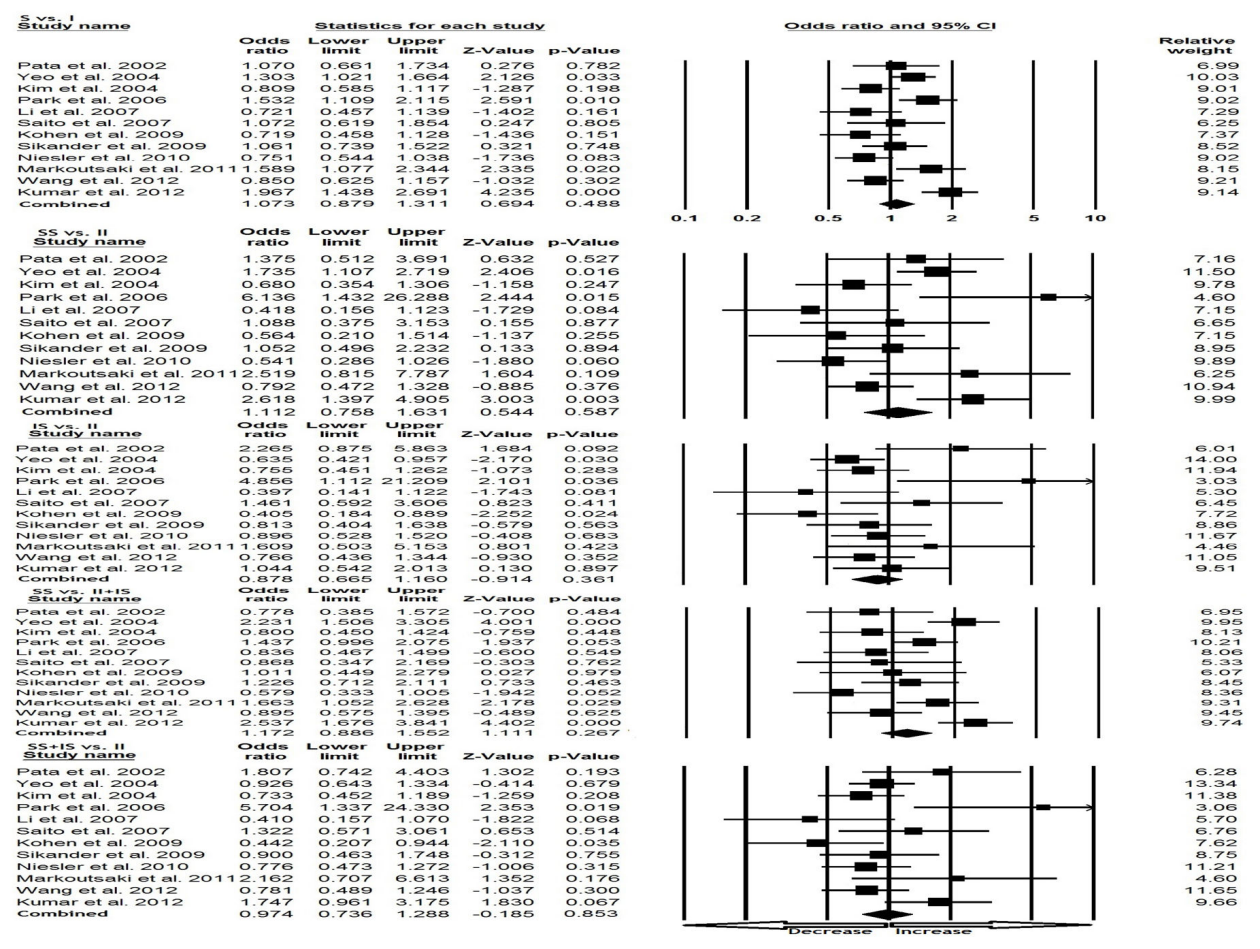
Forest plot of overall IBS risk associated with SLC6A4 (Ins/Del) polymorphism. The squares and horizontal lines correspond to the study-specific OR and 95% CI.

We pooled all the twelve studies together comprising of 2076 controls and 2068 IBS cases, and used random effects model (based on heterogeneity) to evaluate the overall association between the SLC6A4 Ins/Del polymorphism and susceptibility of IBS. On the basis of pooled results, no association has been found under all five genetic models, Variant allele (S vs. I: p=0.488; OR=1.073, 95% CI=0.879 to 1.311), Co-dominant (SS vs. II; p=0.587; OR=1.112, 95% CI=0.758 to 1.631), (IS vs. II; p=0.361; OR=0.878, 95% CI=0.665 to 1.160), Dominant (SS+IS vs. II; p=0.853; OR=0.974, 95% CI=0.736 to 1.288), and Recessive (SS vs. II+IS; p=0.267; OR=1.172, 95% CI=0.886 to 1.552) model, respectively (Figure 1).

### Subgroup analysis of racial descent

We stratified the included studies in to three subgroups by study design and participants’ region or population. This meta-analysis included four studies (668 cases and 650 controls) from American population, three studies (568 cases and 421 controls) from European population, and five studies (832 cases and 1005 controls) from Asian population.

### American population

**Figure 2:**
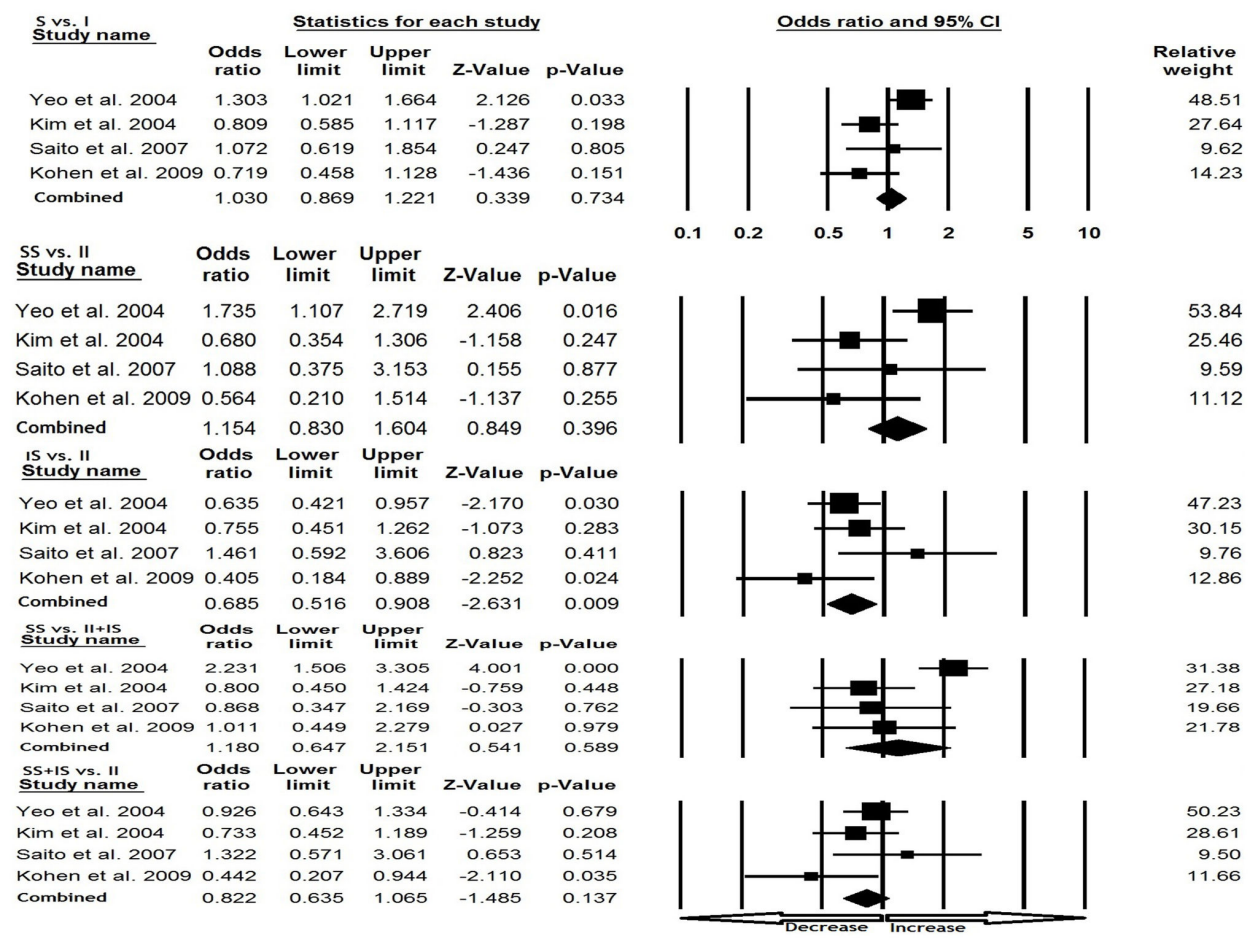
Forest plot for the association between IBS risk and the SLC6A4 (Ins/Del) polymorphism in American population (subgroup analysis).

In American population, heterogeneity was observed in two genetic models [(S vs. I) and (SS vs. II+IS)], so random effect model was applied. However, publication bias did not exist (Table 3). We observed reduced IBS risk under heterozygous comparison (IS vs. II: p=0.009; OR=0.685, 95% CI=0.516 to 0.908). Whereas, no significance was observed in rest of the genetic models, allele (S vs. I: p=0.734; OR=1.030, 95% CI=0.869 to 1.221), homozygous (SS vs. II: p=0.396; OR=1.154, 95% CI=0.830 to 1.604), dominant model (SS+IS vs. II; p=0.137; OR=0.822, 95% CI=0.635 to 1.065) and recessive (SS vs. II+IS; p=0.589; OR=1.180, 95% CI=0.647 to 2.151) comparisons (Figure 2).

**Table 3:** Statistics to test publication bias and heterogeneity for subgroup analysis.

**Comparisons**	**Intercept**	**Egger’s regression analysis**		**Heterogeneity analysis**			**Model used for the meta-analysis**
		**95% CI**	**p-value**	**Q-value**	**P_heterogeneity_**	**I^2^ (%**)	
**Asian population**							
S vs. I	-7.99	-32.57 to 16.59	0.37	21.77	<0.0001	81.63	Random
SS vs. II	1.57	-9.07 to 12.17	0.66	17.72	0.001	77.43	Random
IS vs. II	1.78	-5.26 to 8.83	0.47	7.94	0.093	49.67	Fixed
SS+IS vs. II	-4.30	-19.11 to 10.50	0.42	22.12	<0.0001	81.92	Random
SS vs. II+IS	-0.22	-14.32 to 13.88	0.96	16.22	0.003	73.34	Random
**European population**							
S vs. I	5.48	-108.30 to 119.27	0.65	8.47	0.01	76.39	Random
SS vs. II	5.85	-1.433 to 13.338	0.06	6.32	0.04	68.37	Random
IS vs. II	2.68	-16.03 to 21.39	0.31	3.12	0.21	35.89	Fixed
SS+IS vs. II	1.81	-37.79 to 41.42	0.66	1.26	0.53	<0.0001	Fixed
SS vs. II+IS	-4.60	-41.81 to 32.61	0.36	6.47	0.03	69.12	Random
**American population**							
S vs. I	-2.82	-14.68 to 9.03	0.41	8.18	0.04	63.34	Random
SS vs. II	-2.89	-11.71 to 5.92	0.29	7.72	0.05	61.16	Fixed
IS vs. II	0.95	-9.59 to 11.50	0.73	4.68	0.19	35.97	Fixed
SS+IS vs. II	-0.76	-10.41 to 8.89	0.76	4.42	0.21	32.20	Fixed
SS vs. II+IS	-3.93	-13.35 to 5.47	0.21	10.65	0.01	71.85	Random

### European population

**Figure 3:**
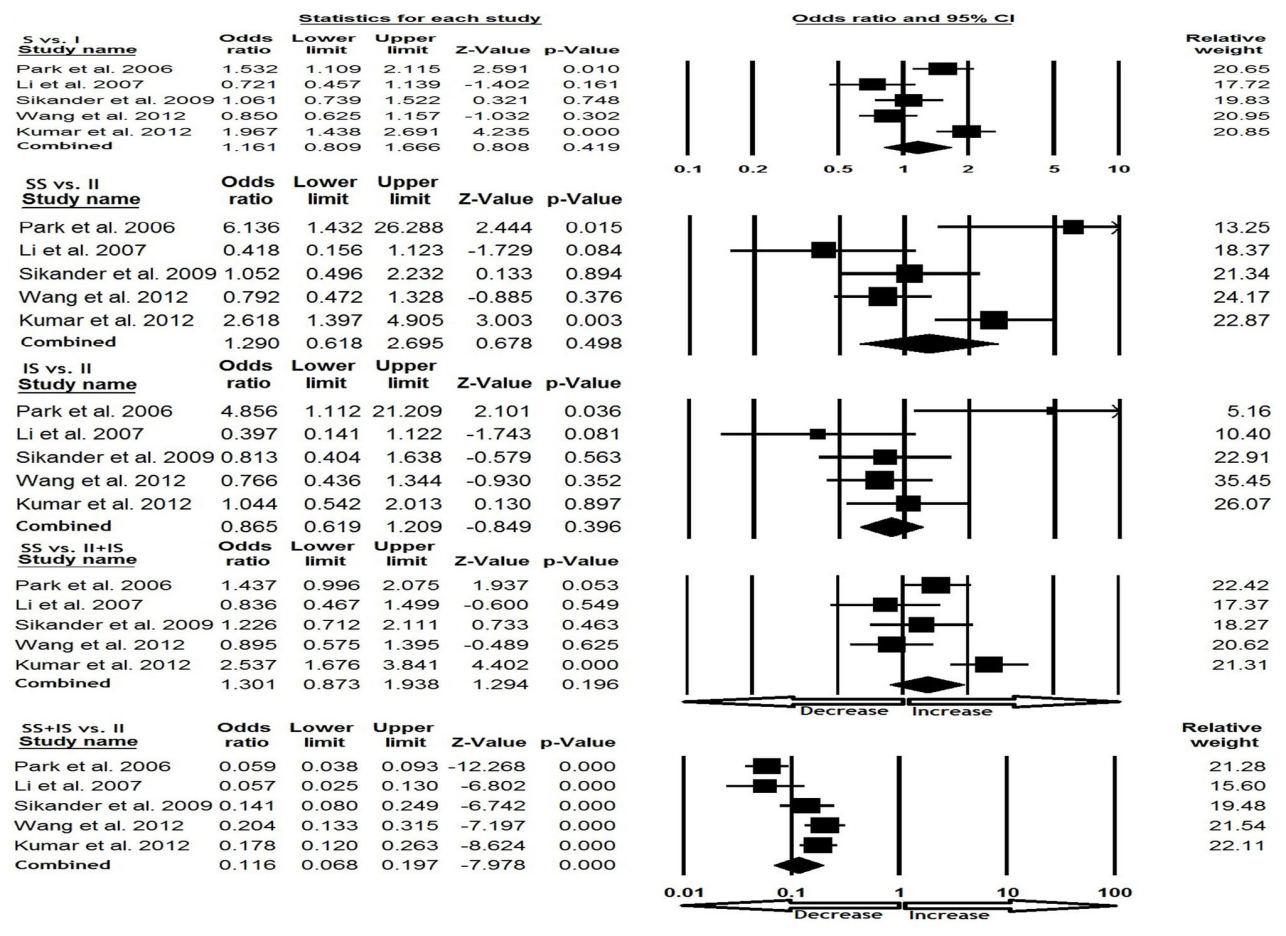
Forest plot for the association between IBS risk and the SLC6A4 (Ins/Del) polymorphism in European population (subgroup analysis).

In European population, random effect model was applied in three [(S vs. I), (SS vs. II) and (SS vs. II+IS)] genetic models based on heterogeneity. However, no publication bias was detected (Table 3). No significant association was found under all genetic models, allele (S vs. I: p=0.791; OR=1.030, 95% CI=0.826 to 1.285), homozygous (SS vs. II: p=0.801; OR=1.127, 95% CI=0.445 to 2.855), heterozygous (IS vs. II: p=0.469; OR=1.172, 95% CI=0.763 to 1.801), dominant (SS+IS vs. II; p=0.850; OR=1.020, 95% CI=0.830 to 1.253), and recessive (SS vs. II+IS; p=0.696; OR=0.905, 95% CI=0.549 to 1.492) model comparisons (Figure 3).

### Asian population

**Figure 4:**
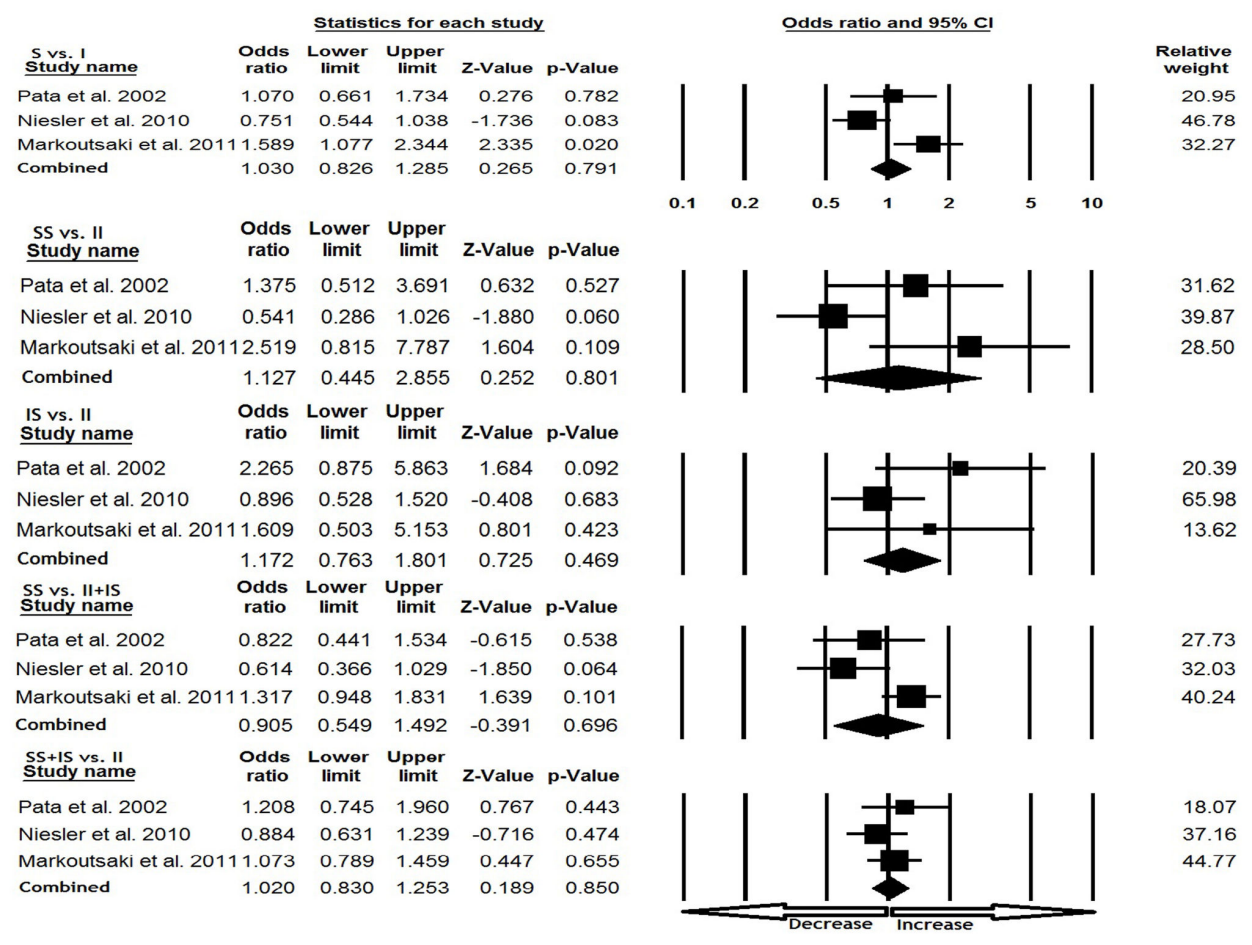
Forest plot for the association between IBS risk and the SLC6A4 (Ins/Del) polymorphism in Asian population (subgroup analysis).

While accessing the Asian population, random effect models were applied for heterogeneity and no publication bias was detected (Table 3). The evaluation of dominant studies revealed reduced risk (SS+IS vs. II; p=0.001; OR=0.116, 95% CI=0.068 to 0.197) of IBS. No significant association was found in rest of the four genetic models (S vs. I: p=0.419; OR=1.161, 95% CI=0.809 to 1.666), homozygous (SS vs. II: p=0.498; OR=1.290, 95% CI=0.618 to 2.695), heterozygous (IS vs. II: p=0.396; OR=0.865, 95% CI=0.619 to 1.209), recessive (SS vs. II+IS; p=0.196; OR=1.301, 95% CI=0.873 to 1.938) (Figure 4).

## Discussion

Despite recent advancements in IBS diagnosis, the prognosis of patients with IBS remains dismal. It is well known that IBS susceptibility is determined not only by the infectious agents, psychosocial and environmental factors, genetic factors also play an important role in their pathogenesis [32,33]. In the recent years, interest in the genetic susceptibility to IBS has led to a growing attention to the study of polymorphisms of genes involved in IBS. As a result, numbers of candidate gene have been investigated to access the probable association between modulations of IBS risk across different population. Till now, a series of studies have been performed to address the association between SLC6A4 (Ins/Del) gene polymorphism and risk of IBS, but the results from different published studies were inconsistent. In order to improve the statistical power and provide the more comprehensive and reliable conclusion, we performed the present meta-analysis to investigate the association between the SLC6A4 (Ins/Del) polymorphism and IBS risk from twelve independent case-control studies, as combining data from many studies has the advantage of reducing random error [34].

Overall analysis of pooled results indicated that SLC6A4 (Ins/Del) polymorphism has no significant association with the IBS risk. Whereas, when we stratified the selected studies by the participants’ region or population, we detected a reduced effect of the polymorphism on IBS risk in American and Asian studies. Previously, Van Kerkhoven et al. performed the meta-analysis and reported no association between SLC6A4 (Ins/Del) polymorphism and IBS susceptibility [35]. However, they warranted for further studies with greater case-control samples possibly due to dearth of sufficient number of published reports in their study. In comparison with earlier report of Van Kerkhoven et al. [35], this study has some major improvements because of inclusion of more number of relevant published studies, which have been published during the past five years.

Alterations in 5-HT signaling, such as, release or reuptake may contribute to chaotic GI function and hypersensitivity reported in IBS patients [36]. Earlier reports have revealed alternations of serotonin metabolism in IBS patients, but findings are remaining inconsistent [13-24]. Coates et al. reported about significant reduction in levels of SERT expression among diarrhea-predominant IBS (IBS-D) patients [37]. However, Dunlop et al. reported raised plasma levels of 5-HT in a similar population [38]. One of larger studies has also demonstrated no differences between SLC6A4 genotype in IBS patients and healthy controls [15].

In addition, IBS pathogenesis is considered to be multifactorial and single genetic variant is usually insufficient to predict the risk of this disease. One important characteristic of this gene polymorphism is that their incidence may vary substantially between different populations and/or ethnicities. Heterogeneity between the studies is very common in the meta-analysis of genetic association studies. While pooling all the eligible data we found inter-study heterogeneity. But after subgroup analysis by participant’s region or population types, the heterogeneity was effectively decreased. Therefore, it can be assumed that the relatively heterogeneity mainly results from differences of population and/or ethnicity. Also, other factors might be involve in this heterogeneity, such as genotype distributions of SLC6A4 (Ins/Del) locus varied between different population and environmental exposures in different case-control studies were not investigated; possibly, these may also influence the genetic susceptibility. Certain limitations were associated with our study, which may affect the results and should be addressed in future studies. For e.g., First, studies published in English language were included; studies indexed by the selected electronic databases were included for the data analysis; it is possible that some relevant articles published in other language and indexed in other databases may have missed. Second, these results are based on unadjusted ORs.

## Conclusion

In conclusion, results of this meta-analysis clearly indicate that SLC6A4 (Ins/Del) polymorphism is associated with reduced risk of IBS in American and Asian population. The importance of this polymorphism as a predictor of the risk of IBS is probably limited and the screening utility of this genetic variant in asymptomatic individuals may not be warranted. The heterogeneity of the IBS population poses challenges for investigators focused on its pathogenesis and therapy. Well-designed large scale association studies incorporated with environmental factors in different populations are needed to validate this association as identified in the present meta-analysis and to investigate the potential gene-gene and gene-environment interaction between SLC6A4 (Ins/Del) polymorphism and susceptibility to IBS.

## Supporting Information

Checklist S1 **PRISMA 2009 Checklist.**
(DOC)

Figure S1 **PRISMA 2009 Flow Diagram: Showing identification and selection of studies for the meta-analysis.**
(TIF)
