# Supplementary figures and images for: A Serotonin Transporter Gene (SLC6A4) Polymorphism Is Associated with Reduced Risk of Irritable Bowel Syndrome in American and Asian Population: A Meta-Analysis
Source: PLoS One. 2013 Sep 19;8(9):e75567. doi: 10.1371/journal.pone.0075567 (PMC3777956; doi:10.1371/journal.pone.0075567)

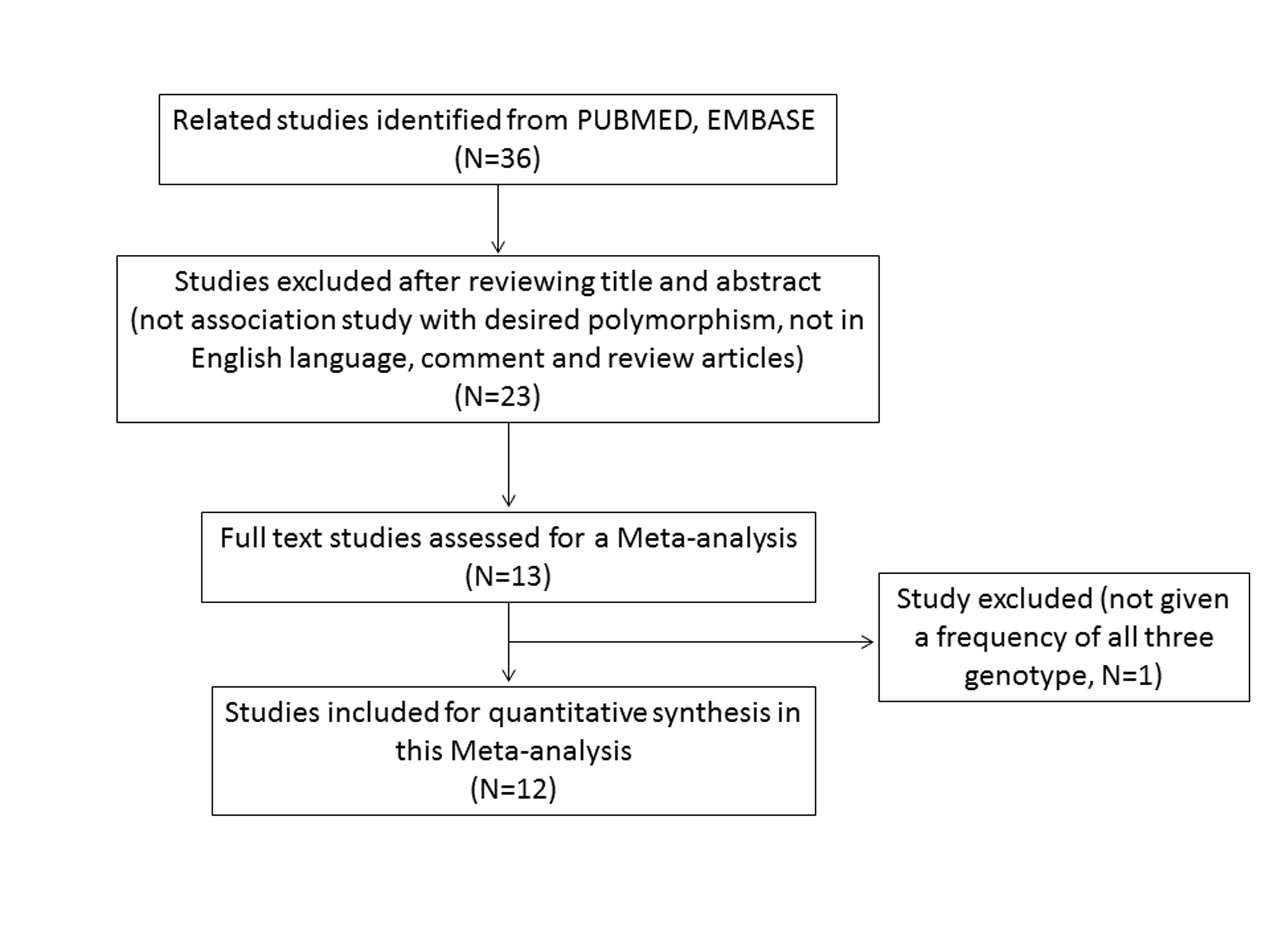

Supplement: Figure S1 — PRISMA 2009 Flow Diagram: Showing identification and selection of studies for the meta-analysis. (TIF) [file pone.0075567.s002.tif]
